# Glycopeptide Hypersensitivity and Adverse Reactions

**DOI:** 10.3390/pharmacy8020070

**Published:** 2020-04-21

**Authors:** Vanthida Huang, Nicola A. Clayton, Kimberly H. Welker

**Affiliations:** 1 Department of Pharmacy Practice, Midwestern University College of Pharmacy-Glendale, Glendale, AZ 85308, USA; 2 Department of Pharmacy Services, UC Davis Health, Sacramento, CA 95817, USA; naclayton@ucdavis.edu; 3 Department of Pharmacy Services, Mercy Gilbert Medical Center, Dignity Health, Gilbert, AZ 85297, USA; kimberly.welker@dignityhealth.org

**Keywords:** dalbavancin, glycopeptides, hypersensitivity, lipoglycopeptides, oritavancin, Redman Syndrome, teicoplanin, telavancin, vancomycin

## Abstract

Glycopeptides, such as vancomycin and teicoplanin, are primarily used in the treatment of methicillin-resistant *Staphylococcus aureus* (MRSA) infections, such as cellulitis, endocarditis, meningitis, pneumonia, and septicemia, and are some of the most commonly prescribed parenteral antimicrobials. Parenteral glycopeptides are first-line therapy for severe MRSA infections; however, oral vancomycin is used as a first-line treatment of *Clostridioides difficile* infections. Also, we currently have the longer-acting lipoglycopeptides, such as dalbavancin, oritavancin, and telavancin to our armamentarium for the treatment of MRSA infections. Lastly, vancomycin is often used as an alternative treatment for patients with β-lactam hypersensitivity. Common adverse effects associated with glycopeptide use include nephrotoxicity, ototoxicity, and Redman Syndrome (RMS). The RMS is often mistaken for a true allergy; however, it is a histamine-related infusion reaction rather than a true immunoglobulin E (IgE)-mediated allergic reaction. Although hypersensitivity to glycopeptides is rare, both immune-mediated and delayed reactions have been reported in the literature. We describe the various types of glycopeptide hypersensitivity reactions associated with glycopeptides and lipoglycopeptides, including IgE-mediated reactions, RMS, and linear immunoglobulin A bullous dermatosis, as well as describe cross-reactivity with other glycopeptides.

## 1. Introduction

The glycopeptide antibiotics, vancomycin and teicoplanin, were discovered in the 1950s and 1990s, respectively [1,2,3]. Newer, long-acting glycopeptides, lipoglycopeptides, including such agents as telavancin, dalbavancin, and oritavancin, have been approved by the U.S. Food and Drug Administration (FDA) within the last decade [4,5,6,7]. Glycopeptide antibiotics have been used to treat gram-positive infections for over five decades. They are commonly used to treat infections caused by methicillin-resistant *Staphylococcus aureus* (MRSA), penicillin-resistant *Streptococcus pneumoniae* (PRSP), and *Clostridioides difficile* [2,8].

In 2011, vancomycin was recommended as the first-line agent for the treatment of MRSA infections in adults and children by the Infectious Disease Society of America (IDSA) [8]. Though vancomycin-resistant enterococci (VRE) and vancomycin-resistant *S. aureus* (VRSA) infections have been noted, vancomycin is still widely used as the empiric treatment of choice for suspected β-lactamase-producing gram-positive infections [9,10]. Common adverse events associated with vancomycin use include the infusion-related reaction known as “red person’s syndrome” or “Redman Syndrome” (RMS), nephrotoxicity, ototoxicity (to a lesser extent), neutropenia, thrombocytopenia, and acute interstitial nephritis (AIN) [11,12,13,14,15,16,17,18,19,20]. Life-threatening immunoglobulin E (IgE)-mediated allergic reactions to vancomycin are rare; however, true allergic reactions may be mistaken for RMS given the overlap in clinical presentation, leading to confusion, incorrectly challenging patients with true IgE-mediated reaction, or the use of alternative agents such as daptomycin or linezolid in patients who experience RMS. Other immune-mediated reactions, such as linear immunoglobulin A (IgA) bullous dermatosis (LABD), Stevens-Johnson syndrome (SJS), and toxic epidermal necrolysis (TEN), have also been observed with vancomycin use [21,22,23]. Hypersensitivity reactions are summarized based on the type in Table 1. The objective of this review is to describe the variety of hypersensitivity reactions known to occur with glycopeptide use from mild (RMS) to severe reactions (IgE-mediated and LABD).

## 2. Immunoglobulin E (IgE)-Mediated Reactions

### 2.1. Incidence

There are two types of anaphylactoid hypersensitivities reported with glycopeptides and lipoglycopeptides, especially vancomycin; RMS and anaphylaxis. The abundance of data supporting the hypersensitivity will be from vancomycin as it has been on the market for over five decades. The IgE-medicated reactions occur when it binds to Fc-epsilon-RI receptors, and upon exposure to the allergen, it activates mast and basophil cells to release multiple mediators, enzymes, and cytokines that trigger signs and symptoms of anaphylaxis [28]. Anaphylaxis is a rare but serious immunologically mediated reaction involving drug-specific IgE antibodies [20,29,30,31,32,33]. The reactions noted due to vancomycin anaphylaxis with demonstrable antibiotic-specific IgE are clinical manifestations described below [29]. The incidence of anaphylaxis is not well known; however, in a study by Minhas and colleagues, approximately 10% (7/71 identified cases of vancomycin hypersensitivity reactions) were IgE-mediated hypersensitivity reactions [20]. They also demonstrated that 57% of the cases were female, with a median age of 43 years. Most of the patients presented with hypotension, respiratory symptoms, prior exposure to vancomycin, and skin findings. The most significant risk associated with an anaphylactic reaction to vancomycin is multiple previous vancomycin exposures. As with other hypersensitivity reactions, true vancomycin hypersensitivity does not occur upon first exposure but rather subsequent exposure due to antibiotic-specific IgE antibodies. However, IgE-mediated reactions can occur after the first exposure. Case reports summary of IgE-mediated in both exposures to intravenous (IV) and oral (PO) vancomycin are listed in Table 2 and Table 3.

Symptom onset occurs within a median of two minutes during infusion of the dose in patients who have anaphylactic reactions [20]. The clinical manifestation includes the following: angioedema, bronchospasm, respiratory distress, generalized pruritus, hypotension, urticaria, tachycardia, nausea and vomiting, and lightheadedness [24,29]. The diagnosis can be difficult because one has to differentiate between severe RMS and anaphylaxis based on clinical presentations. Treatment for anaphylaxis consists of immediate discontinuation of the glycopeptide and immediate treatment with epinephrine, antihistamines, or corticosteroids as indicated [20,29,30]. If no other therapeutic options are available for the treatment of the infection, the glycopeptide can be desensitized by introducing fractional doses over an extended period.

### 2.2. Desensitization

Patients can be desensitized to glycopeptides if no equivalent therapy is available. However, in the last decade, we have an abundance of newly approved anti-MRSA therapy where vancomycin desensitization has become obsolete. Alternative oral and parenteral agents are summarized in Table 1. The desensitization protocol is a process that allows the patient to receive an uninterrupted course of the medication safely. Desensitization is a process of administering vancomycin at a low dose and slowly increasing doses to eventually render mast cells unresponsive to the medication. It alters the immune response to the antibiotic to ultimately provide a temporary tolerance [40]. Desensitization is implemented when the need for medication outweighs the risk of the reaction during the procedure where the patient has failed therapy, or an alternative is not available. It is essential to monitor the patient closely for an allergic reaction, with treatment or desensitization readily available. Desensitization is not permanent, as sensitization may recur once regular exposure to the drug is stopped. There are several parenteral vancomycin desensitization protocols that have been published (Table A1 in Appendix A) [41,42]. Both rapid and slow desensitization protocols require premedication. In many cases, if a patient does not tolerate the rapid desensitization protocol, or if it is a refractory case, then a slow desensitization protocol may be indicated [30,41,42,43,44,45]. Since vancomycin desensitization may be considered obsolete, we will only briefly describe the protocol for the completion of a therapeutic option.

A rapid desensitization protocol was first described by Lerner and colleagues [42]. The study challenged a female patient with recurrent staphylococcal skin abscess. The protocol calls for the patient to receive both an antihistamine (diphenhydramine 50 mg) and a corticosteroid (hydrocortisone 100 mg) 15 min prior to the vancomycin infusion protocol [42,46]. The initial dose of vancomycin was slowly administered at a rate of 0.5 mL/min and gradually increased to 5 mL/min for the highest concentrations. Four concentrations of vancomycin were prepared from a 10 mL standard solution of 500 mg in 250 mL (2 mg/mL) normal saline or dextrose 5% water (D5W). The standard solution was serially diluted by tenfold at 1:10 (0.2 mg/mL), 1:100 (0.02 mg/mL), 1:1000 0.002 mg/mL), and 1:10,000 (0.0002 mg/mL) into 100 mL saline/D5W to produce four 100mL bags. The lowest concentration (0.0002 mg/mL) was infused first, followed the lowest remaining concentration sequentially until all were infused (takes about four hours). If the patient tolerates the escalation protocol, then subsequent full vancomycin doses can be given to the patient.

A slow desensitization protocol was described by Lin and colleagues [41]. The authors describe a patient with MRSA bacteremia who was being treated with vancomycin but was inadvertently discontinued for 24 h. The patient developed an anaphylactic reaction (hypotension, redness, diffuse pruritus) within five minutes of vancomycin re-administration. This reaction was confirmed positive by skin-prick testing. The patient developed a second reaction a week later. Vancomycin desensitization was initiated two weeks after the initial reaction occurred. The protocol called for premedication with diphenhydramine 50 mg intramuscularly every six hours and ranitidine 150 mg every six hours concomitantly. Vancomycin 0.5 mg in 500 mL of normal saline was administered over five hours on day 1. Subsequent days 2, 3, 4, 5, and 6, the vancomycin dose was increased to 5 mg, 50, 50, 250, and 500 mg in 500 mL normal saline, respectively. On day 7, vancomycin was administered at 500 mg every six hours. The following day, vancomycin was administered at the full dose of 1 gram every 12 h, and both antihistamines were discontinued. After desensitization, the patient was discharged without consequences after six weeks of treatment with vancomycin. This slow dosing strategy was chosen based on a study by Polk and colleagues who demonstrated that altering the dose and frequency reduced the hypersensitivity reactions [27]. The study used half the vancomycin dose (500 mg vs. 1 g) while increasing the frequency from every 12 h to every 6 h. The authors demonstrated that receiving vancomycin 500 mg every six hours has less chance of developing hypersensitivity reactions.

There is also a desensitization protocol for oral vancomycin. Although oral vancomycin has little systemic absorption, and thus, fewer IgE-mediated reactions to be expected, there have been case reports of anaphylaxis following the administration of oral vancomycin to treat *C. difficile* infections [37,38]. Patients with active *C. difficile* infections may have a non-intact gastrointestinal mucosa leading to increased absorption [37]. Therefore, caution should be used in patients with a history of anaphylaxis to intravenous vancomycin administration when administering oral vancomycin for the treatment of *C. difficile* infections [1,47].

## 3. Delayed Hypersensitivity Reactions

Immunoglobulin G (IgG)- or immunoglobulin M (IgM)-mediated hypersensitivity reactions, also known as Type II reactions, can occur following vancomycin therapy. Thrombocytopenia is more commonly seen than hemolytic anemia or neutropenia and usually resolves within 72–96 h of discontinuing vancomycin [48].

Delayed hypersensitivity reactions have been documented following vancomycin administration and usually occur 48–96 h following exposure in previously sensitized patients or up to 14 days later in non-sensitized patients [20,48]. In particular, vancomycin-induced LABD comprises 46% of drug-induced LABD cases, but SJS, TEN, and drug rash with eosinophilia and systemic symptoms (DRESS) have also been documented [21,22].

### 3.1. Linear Immunoglobulin A Bullous Dermatosis

Vancomycin is the most common antibiotic-associated with LABD; see case report summaries in Table 4. Clinically characterized by the appearance of multiple small itchy bullae within annular erythema on the entire body, LABD risk factors include male sex and age greater than 68. Skin lesions typically appear 1 to 21 days upon receipt of vancomycin, and symptoms typically disappear after vancomycin is discontinued, but it can take up to 60 days for complete resolution [23]. Diagnosis of LABD may be complicated and difficult to differentiate from other severe cutaneous reactions as 42% of patients have mucosal involvement, 20% have lesions mimicking TEN, and 21% have eosinophil infiltrates [49]. LABD relies on direct immunofluorescence (DIF) testing showing linear deposition of immunoglobulin IgA along the basement membrane of the epidermis, present in up to 80% of cases, for diagnosis; however, cases of LABD with an initial negative DIF followed by repeat positive testing have been noted [50]. Typically, the only cessation of vancomycin is necessary for the resolution of symptoms; adjunctive topical corticosteroids may also be initiated. Successful completion of a treatment course of vancomycin has also been documented with concomitant systemic corticosteroids [23].

Kakar and colleagues reported a case of LABD presenting as TEN [25]. An elderly female presented to a burn unit for evaluation of possible toxic epidermal necrolysis. She had multiple comorbidities, including diabetes, atrial fibrillation, end-stage renal disease, and Crohn’s disease. The patient initially had vesicles on her soft pallet, which progressed to bullae on her back within two days. Direct immunofluorescence was performed, showing linear IgA deposits. A biopsy was performed showing subepidermal bulla with neutrophilic infiltrate. The biopsy did not show epidermal necrosis. The patient was previously admitted to another facility two weeks prior, where she was treated for acute cholecystitis complicated by fistula and sepsis. She received multiple antibiotics, including vancomycin and piperacillin/tazobactam; the duration of antibiotics was not specified. Skin sloughing over 40% of her body surface area was described, and, based on the patient’s worsening clinical status, the family decided on comfort care. Findings consistent with LABD: Direct immunofluorescence showed linear IgA deposition along the mucous membrane, bullae, and erythematous patches. This patient also had mucous membrane involvement and palm/sole involvement. The authors of this case concluded that mucous membrane involvement may or may not be present in LABD mimicking TEN.

### 3.2. Drug Rash with Eosinophilia and Systemic Symptoms

As of 2017, 23 cases of DRESS associated with vancomycin use have been reported; see some case summaries in Table 5. Typically, patients present with a skin rash accompanied by eosinophilia, fever, atypical leukocytosis, and multiple organ failures, including the kidneys, liver, and lungs [51,52]. Mortality rates have been documented as high as 10%, and, unlike other precipitants, vancomycin is particularly associated with an increased risk of renal involvement [53]. Seventy-four percent (74%) of DRESS cases are associated with antibiotics, with the most commonly implicated antibiotics being vancomycin (39%), β-lactams (23%), fluoroquinolones (4%), tetracyclines (4%) and sulfonamides (3%) [54]. The hallmark of DRESS, in contrast to the other severe cutaneous reactions noted above, is the long latency period and the lack of mucosal involvement [55,56]. Symptoms usually appear two to six weeks after initial drug exposure. DRESS has been noted in patients receiving parental vancomycin and with concomitant surgically implanted vancomycin impregnated bone cement; however, symptoms resolved after the parenteral vancomycin was discontinued even though the bone cement was not surgically removed [57]. Prompt discontinuation of the offending agent and pulsed corticosteroids are considered the mainstay of treatment [55]. A rapid taper of corticosteroids can lead to the recurrence of DRESS and rehospitalization; therefore, a slow taper over four to six weeks after the resolution of rash is suggested [56]. Recent literature suggests a strong association of the HLA-A*32:01 alleles in patients of European ancestry who develop DRESS while on vancomycin. The risk of DRESS approaches 20% at four weeks of therapy in those carrying the HLA-A*32:01 allele [58]. While the current HLA allele testing turnaround time is often too prolonged to lead to clinically relevant data, the long latency period of DRESS may allow for testing after initiation of vancomycin in patients who will require a long treatment course and a cost effective HLA gene panel with a 48-h turnaround time is in development [54,58].

### 3.3. Vancomycin-Induced Acute Interstitial Nephritis

Acute interstitial nephritis is rare and believed to be a hypersensitivity reaction, especially with vancomycin; selected case reports are shown in Table 6. It is most commonly seen in β-lactams antibiotics. There were 11 cases reported in the literature due to vancomycin [14,20,59,60,61]. It is believed that AIN results when the medication triggers an immune response by forming circulating complexes that deposit in the interstitium [62,63]. It typically occurs within 3–5 days of post-secondary exposure but takes weeks from first exposure [64]. Based on the 11 patient cases, the onset of vancomycin-induced AIN is approximately 7–33 days post administration of the offending agents. The clinical signs and symptoms seen with AIN are fever, eosinophilia, a maculopapular rash with sudden renal impairment, in addition to flank tenderness, hematuria, arthralgia, pyuria, and proteinuria [62,63,64]. Renal biopsy is necessary to confirm the diagnosis of AIN. Treatment of AIN involves identifying and immediately discontinuing the offending agent [64]. Short-term corticosteroids can be considered for the management of AIN until the renal function has recovered.

## 4. False Hypersensitivity Reaction: Redman Syndrome

### 4.1. Incidence

The occurrence of RMS associated with the infusion of vancomycin is well known and is often documented as an allergy to vancomycin but should be more appropriately characterized as a “pseudoallergy”, summarized in Table 7. Early reports of RMS characterized the infusion reaction as an anaphylactoid response and noted that 50–90% of patients who received vancomycin had a reaction, though most were mild [67]. The incidence of RMS has been markedly reduced by the additional purification of the commercially available drug product and by slowing the infusion time to no less than one hour [68]. RMS is caused by non-IgE-mediated mast cell degranulation with histamine release [55]. Clinical symptoms include erythema, flushing, pruritus, and, in some instances, hypotension and angioedema that are noted after the rapid infusion of vancomycin, but laryngeal edema is not usually noted [11,69]. Tachyphylaxis usually occurs rapidly after the first dose, and subsequent infusions are well tolerated [27]. RMS occurs due to the direct stimulation of mast cells leading to histamine release rather than from the production of antibodies. So, unlike a true allergy, RMS can occur without prior exposure to vancomycin [48,67].

### 4.2. Risk Factors

A rapid infusion rate is the most common risk factor for developing RMS, though large doses relative to body weight have also been associated with RMS. Symptoms typically begin with pruritus starting on the top of the head or back of the neck and evolve into facial erythema, which can extend to the chest and back. Symptoms usually begin within 20 to 45 min of the start of the infusion and begin to resolve a few minutes after the infusion is stopped with a total resolution of symptoms within one hour. More severe symptoms, such as facial edema and hypotension, can occur but are less common [69]. Patients with severe symptoms who do not respond quickly to the cessation of vancomycin and an antihistamine may be given via intravenous fluids and corticosteroids [69]. Patients who experience a severe reaction to the first dose are more likely to develop a reaction during subsequent doses, though usually the subsequent reactions are more mild than the initial reaction [67].

Though available data are limited and the published studies have a small sample size, RMS does not appear to occur in patients who receive a continuous infusion since it is more likely with intermittent infusion [70,71]. Also, reducing the infusion rate and premedication with diphenhydramine or hydroxyzine allows for greater tolerance of vancomycin infusions by competitively binding to H_1_-receptors [67]. Clear documentation of an IgE-mediated reaction versus RMS with subsequent tolerance of vancomycin is essential. Since vancomycin is widely used for gram-positive infections, specifically MRSA, the alternatives can be a broader spectrum than necessary or have a less favorable susceptibility profile.

## 5. Cross-Reactivity with Other Glycopeptides

### 5.1. Teicoplanin

Teicoplanin, currently unavailable in the United States, is the most well studied of the glycopeptides. While teicoplanin is structurally similar to vancomycin, RMS and the accompanying clinical symptoms have only rarely been noted, even at high doses of 30 mg/kg and patients who experience RMS with vancomycin infusions have been switched to teicoplanin without recurrence of RMS [11]. IgE-mediated allergic reactions with cross-reactivity between vancomycin and teicoplanin have been documented [72]. In one study, 53.8% of patients who experienced leukopenia, thrombocytopenia, and rash secondary to vancomycin administration had a subsequent adverse drug reaction to teicoplanin [73]. Notably, there are also case reports of DRESS, SJS, and glycopeptide-induced vasculitis associated with vancomycin use that was further exacerbated by subsequent teicoplanin administration and only resolved after the discontinuation of teicoplanin [72,74,75].

### 5.2. Telavancin

Telavancin received approval from the FDA in 2009 for the treatment of complicated skin and soft tissue infections. As a semi-synthetic derivative of vancomycin, it is unknown if patients with hypersensitivity reactions to vancomycin will experience cross-reactivity to telavancin, but serious and sometimes fatal reactions have been observed in post-marketing surveillance. RMS-like infusion reactions have been noted, and as such, telavancin should be infused over no less than 60 min [76]. One study in pediatric cystic fibrosis patients who received telavancin suggested that a previous intolerance to vancomycin such as RMS, should not prohibit a trial of telavancin but that caution should be used in patients with a history of IgE-mediated reactions as cross-reactivity was observed [77].

### 5.3. Dalbavancin and Oritavancin

Dalbavancin and oritavancin are semi-synthetic derivatives of vancomycin and teicoplanin, respectively [68]. Both dalbavancin and oritavancin were FDA approved in 2014. Like telavancin, dalbavancin and oritavancin contain lipophilic side chains that allow for increased ability to anchor into the binding sites on the growing cell wall. Unlike vancomycin, these lipoglycopeptides are known to be rapidly bactericidal due to their increased potency. Though no cross-reactivity is noted between vancomycin and dalbavancin or oritavancin, their prolonged half-lives of 257 and 195 h, respectively, raise concerns of an allergic reaction that must be controlled for a much longer time period [5]. Though cross-reactivity between vancomycin and dalbavancin or oritavancin is not well known, caution should be used when these agents are necessary for use in patients with a history of anaphylaxis to other glycopeptides and should only be used if the benefit outweighs the risk [78,79]. Although hypersensitivity to prior glycopeptides is not a contraindication to dalbavancin or oritavancin use, a thorough patient history is necessary, and patients should be carefully monitored during the infusion for signs of a repeat reaction. In Phase 3, acute bacterial skin and skin structure infection clinical trials for oritavancin, the median onset of hypersensitivity reactions in oritavancin-treated patients was 1.2 days and the median duration of these reactions was 2.4 days [78]. Reactions may not be immediate, but they may persist; therefore, prolonged observation will be necessary.

An infusion-related reaction similar to RMS is noted with both dalbavancin and oritavancin. In clinical trials for oritavancin, 1.9% of patients who received oritavancin had an infusion site reaction compared to 3.5% of patients who received vancomycin. Due to this infusion-related reaction, oritavancin should be infused over three hours. Symptoms typically resolve quickly when the infusion rate is reduced or discontinued [78]. In dalbavancin clinical trials, rash and pruritus occurred in 2.7%, and 2.1% of patients receiving dalbavancin and 2.4% and 3.3% of patients receiving vancomycin, respectively [80]. Dalbavancin should be infused over no less than 30 min to avoid the risk of developing RMS [79]. When infused over 30 min, infusion-related reactions were no more frequent than those associated with vancomycin administered over a period of 120 min [81,82]. Given the recent approval of both dalbavancin and oritavancin, the rate of adverse events, including both RMS and true anaphylaxis, can only be established after more extensive post-marketing clinical use.

## 6. Conclusions

Glycopeptides have been in relatively widespread use since the 1950s for the treatment of multidrug-resistant gram-positive infections and they are commonly used as a beta-lactam alternative in patients with an IgE-mediated allergic reaction to beta-lactams. Currently, vancomycin is the first-line treatment recommended by the IDSA for severe MRSA and *C. difficile* infections. While true anaphylaxis after vancomycin exposure is rare and requires prior exposure, the pseudoallergic infusion reaction known as RMS is common and may occur during any administration of vancomycin. Patients who experience RMS who require subsequent vancomycin administration often experience tachyphylaxis and have milder, if any, reaction during subsequent exposure. For patients who experience a true IgE-mediated reaction to vancomycin and in whom no other treatment choice is appropriate, desensitization may be attempted. There are currently two accepted desensitization protocols: a rapid protocol and a slow protocol. However, currently, we have many alternative agents as treatment options that vancomycin desensitization has become nearly obsolete.

Severe skin rashes due to delayed hypersensitivity reactions have been documented with vancomycin use. Linear IgA bullous dermatosis is the most common, but other types of delayed hypersensitivity have been observed, such as DRESS. The mainstay of treatment is to discontinue the offending agent and initiate corticosteroid therapy. Data regarding cross-reactivity is limited, and caution should be used when alternative glycopeptides or lipoglycopeptides are used. Data is limited regarding glycopeptides other than vancomycin, but all glycopeptides have the possibility of infusion-related reactions, which can typically be mitigated by slowing down the infusion rate.

## Figures and Tables

**Table 1:** Summary of type of hypersensitivity reactions [24,25,26,27].

Reaction Type	Pathogenesis	Median Time-To-Onset	Clinical Presentation	Management Strategies
**IgE-mediated hypersensitivity**	Type I hypersensitivity: It is immunologically mediated with drug-specific IgE antibodies. Most common with multiple prior exposures	Reaction occurs in minutes typically during vancomycin infusion	Angioedema, pruritus, hypotension, urticaria, tachycardia, nausea, and vomiting	Discontinuation of vancomycin, immediate receipt of epinephrine, antihistamines, or corticosteroids
**Delayed hypersensitivity reaction**	Type II delayed hypersensitivity: IgG- or IgM-mediated	7 to 14 days after vancomycin administration	Thrombocytopenia, hemolytic anemia, neutropenia	Discontinuation of vancomycin as soon as possible upon diagnosis
**Linear IgA Bullous Dermatosis (LABD)**	Type IV delayed-hypersensitivity: Linear disposition of IgA along basement membranes of the epidermis	1 to 21 days after vancomycin administration	Small itchy bullae, possible eosinophil infiltrates	Discontinue vancomycin, topical corticosteroids
**Drug rash with eosinophilia and systemic symptoms (DRESS)**	Type IV delayed-hypersensitivity: Eosinophilic activation and inflammatory cascade	2 to 6 weeks after initial drug exposure	Skin rash, fever, atypical leukocytosis, multiple organ failure including kidneys, liver, and lungs	Discontinue vancomycin, pulsed corticosteroids with a slow taper over 4–6 weeks
**Red Man’s Syndromes (RMS)**	Non-IgE-mediated mast cell degranulation with histamine release	Can occur without prior exposure; 20–45 min from the start of infusion; Subsequent infusions likely to be better tolerated	Erythema, flushing, pruritus from top of head or back which can extend to chest and back, hypotension, angioedema	Antihistamine; Resolution of symptoms within an hour of vancomycin being stopped; For severe symptoms, intravenous fluids, and corticosteroids

**Table 2:** Immunoglobulin E (IgE)-mediated reactions case reports.

References	Reactions to VAN	Treatment Patient Received	Allergy Confirmation
Otani IM et al. [34]	Inability to ventilate, hypotension, erythematous flushed skin	IV epinephrine (drip), hydrocortisone, diphenhydramine, albuterol inhalation	Positive skin test
Hwang MJ et al. [35]	Severe prickling sensation, pruritus, urticarial rash, throat tightness	IM epinephrine, dexamethasone, IV antihistamine (unspecified)	Previous exposure flushing, pruritus
Hassaballa H et al. [31]	Pruritus, nausea, hypotension, emesis, tongue swelling	Intubation, epinephrine, hydrocortisone, diphenhydramine	No allergy confirmation
Chopra N et al. [29]	Difficulty breathing, wheezing, hypoxemia, pruritus, erythema entire body	Diphenhydramine	Desensitization to VAN
Knudsen JD et al. [32]	Angioedema, increased HR, fever, anxiety	antihistamine	Histamine release test positive with exposure to VAN/teicoplanin (IgE-mediated)

VAN: vancomycin. IV: intravenous. IM: intramuscular. MRSA: methicillin-resistant S. aureus. HR: heart rate.

**Table 3:** Case reports of reactions to oral vancomycin.

Reference	Reactions to VAN	Treatment Patient Received	Allergy Confirmation	Risk Factors for Systemic Absorption, Pertinent MH
Laehn S et al. [36]	Hives	Unspecified	Desensitization with PO VAN;	Not specified
Baumgartner LJ et al. [37]	Urticarial rash	Unspecified histamine receptor antagonists	Naranjo adverse reaction probability 5	Diverticulitis
Bosse D et al. [38]	Throat tightness, dyspnea, tachycardia, face/laryngeal erythema	IM epinephrine, diphenhydramine, methylprednisolone, ranitidine, saline 1-liter bolus	Reaction with IV VAN exposure	Cystic fibrosis, lung transplant
Mahabir S et al. [39]	Rash developed following IV VAN, PO VAN not given before desensitization	Antihistamine, hydrocortisone following IV VAN	Reaction with IV VAN exposure	Renal impairment, bowel inflammation

VAN: vancomycin. MH: medical history. IV: intravenous. PO: oral. CDI: Clostridioides difficile infection. IM: intramuscular.

**Table 4:** Case reports of linear immunoglobulin A (IgA) bullous dermatosis (LABD).

References	Patient Age	Indication for VAN	Reactions to VAN	Treatment Patient Received	Timeline of Reaction Occurrence
Winn AE et al. [50]	74-year-old female	Skin and soft tissue infection	Erythematous, edematous plaques on neck, trunk, shoulders	Antibiotics discontinued	4 days after initiation of VAN
Zenke Y et al. [23]	62-year-old male	MRSA bacteremia and endocarditis	Erythema on the trunk; bullae on axillae, chest, thighs, buttocks; elevated serum IgA	VAN continued, systemic prednisolone initiated, skin lesions resolved	10 days after initiation erythema occurred, 12 days after erythema bullae developed

VAN: vancomycin. MRSA: methicillin-resistant *S. aureus*.

**Table 5:** Case reports of drug rash with eosinophilia and systemic symptoms (DRESS).

References	Reactions to VAN	Treatment Patient Received	Timeline of Reaction Occurrence
Chamorro-Pareja N et al. [52]	Pruritic rash, facial angioedema, neutrophilia, eosinophilia	VAN discontinued, antihistamines, corticosteroids	Approximately 3 weeks
Wilcox O et al. [56]	Fever, chills, shortness of breath, neutrophilia	VAN discontinued, systemic corticosteroids	Approximately 3 weeks
Webb PS et al. [53]	Rash, AKI, eosinophilia	VAN discontinued, hemodialysis, systemic corticosteroids	Approximately 1 week
Guner MD et al. [57]	Fever, rash, eosinophilia	VAN discontinued, topical/systemic corticosteroids	Approximately 4 weeks
Guner MD et al. [57]	Fever, rash, eosinophilia, increased serum creatinine, increased AST/ALT	VAN discontinued, topical/systemic corticosteroids	Approximately 3 weeks
Marik PE et al. [55]	Maculopapular rash, fever, eosinophilia, increased serum creatinine	VAN discontinued, systemic corticosteroids	Approximately 4 weeks

VAN: vancomycin. IV: intravenous. ALT: alanine aminotransferase. AST: aspartate aminotransferase.

**Table 6:** Case reports of vancomycin-induced acute interstitial nephritis.

References	Reactions to VAN	Treatment Patient Received	Timeline of Reaction Occurrence
Htike NL et al. [65]	Malaise, elevated serum creatinine, eosinophils observed from renal biopsy. Biopsy confirmed ATN/AIN	Prednisone	History of RMS with prior VAN use. In this episode, VAN × 1 week. Serum creatinine returned to baseline after 4 weeks
Hong S et al. [66]	Pruritic rash, fever, elevated serum creatinine, elevated eosinophilia, elevated IgE titers, renal biopsy confirmed AIN	Methylprednisolone, prednisone, diphenhydramine, cyclosporine, mycophenolate, renal replacement therapy	Received VAN × 1 month. Renal function improved after several months
Plakogiannis R et al. [63]	Elevated eosinophilia level, elevated serum creatinine, rash	Topical corticosteroids	Received VAN with ceftriaxone × 4 days Renal function returned to baseline
	Elevated eosinophilia level and elevated serum creatinine	No corticosteroids given	Received VAN with ceftriaxone × 1 month. Renal function improved after several weeks

VAN: vancomycin. ATN: acute tubular necrosis. AIN: acute interstitial nephritis.

**Table 7:** Summary of Redman Syndrome.

Clinical Symptoms	Key Principles to Avoid Red Man’s Syndrome
- Erythema - Flushing - Pruritus - Hypotension	- Slow infusion (no more than 1 gram over 1 h) - Premedication with diphenhydramine or hydroxyzine

## Appendix A

**Table A1:** Oral vancomycin desensitization protocol.

Dose Number	Dose (mg)
1	0.025
2	0.05
3	0.1
4	0.2
5	0.4
6	0.8
7	1.6
8	3.2
9	6.0
10	12.5
11	25
12	50
13	100
14	200
15	400
16	500

Adapted from Laehn N, et al. Each dose should be administered via nasogastric tube given 20 min apart starting at 0.025 mg and escalating up to 500 mg with a total of 16 increasing doses, 500 mg given 6 h after last dose.
